# Targeting Inflammatory T Helper Cells *via* Retinoic Acid-Related Orphan Receptor Gamma t Is Ineffective to Prevent Allo-Response-Driven Colitis

**DOI:** 10.3389/fimmu.2018.01138

**Published:** 2018-05-25

**Authors:** Vera Buchele, Benjamin Abendroth, Maike Büttner-Herold, Tina Vogler, Johanna Rothamer, Sakhila Ghimire, Evelyn Ullrich, Ernst Holler, Markus F. Neurath, Kai Hildner

**Affiliations:** ^1^Department of Medicine 1, University Hospital Erlangen, University of Erlangen-Nuremberg, Kussmaul Campus for Medical Research, Erlangen, Germany; ^2^Department of Nephropathology, Institute of Pathology, University Hospital Erlangen, Erlangen, Germany; ^3^Department of Medicine 5, University Hospital Erlangen, University of Erlangen-Nuremberg, Erlangen, Germany; ^4^Department of Pediatric Stem Cell Transplantation and Immunology, Childrens Hospital, Johann Wolfgang Goethe University, Frankfurt, Germany; ^5^Department of Hematology and Oncology, Regensburg University Hospital, Regensburg, Germany

**Keywords:** retinoic acid-related orphan receptor gamma t, interleukin-23 receptor, T helper 17 cells, granulocyte-macrophage colony-stimulating factor, basic leucine zipper transcription factor ATF-like, intestinal graft-versus-host disease, colitis

## Abstract

Intestinal graft-versus-host disease (GvHD) is a life-threatening, inflammatory donor T cell-mediated complication of allogeneic hematopoietic stem cell transplantation (allo-HSCT). In the light of the reported efficacy of interleukin-23 (IL-23)-blockade to mitigate syngeneic intestinal inflammation in inflammatory bowel disease patients, targeting IL-23 and thereby interleukin-17a (IL-17a) producing T helper (Th17) cells as the T cell subset assumed to be mostly regulated by IL-23, has emerged as a putatively general concept to harness immune-mediated mucosal inflammation irrespective of the underlying trigger. However, the role of Th17 cells during allo-response driven colitis remains ambiguous due to a series of studies with inconclusive results. Interestingly, we recently identified granulocyte-macrophage colony-stimulating factor (GM-CSF^+^) T cells to be promoted by interleukin-7 (IL-7) signaling and controlled by the activating protein-1 transcription factor family member basic leucine zipper transcription factor ATF-like (BATF) as critical mediators of intestinal GvHD in mice. Given the dual role of BATF, the contribution of IL-23-mediated signaling within donor T cells and bona fide Th17 cells remains to be delineated from the regulation of GM-CSF^+^ T cells in the absence of BATF. Here, we found in a complete MHC class I-mismatched model that genetic inactivation of the IL-23 receptor (IL-23R) or the transcription factor retinoic acid-related orphan receptor gamma t (RORγt) within donor T cells similarly ablated Th17 cell formation *in vivo* but preserved the T cells’ ability to induce intestinal GvHD in a compared to wild-type controls indistinguishable manner. Importantly, RORγt-independent manifestation of intestinal GvHD was completely dependent on BATF-regulated GM-CSF^+^ T cells as BATF/RORγt double-deficient T cells failed to induce colitis and the antibody-mediated blockage of IL-7/IL-7R interaction and GM-CSF significantly diminished signs of intestinal GvHD elicited by RORγt-deficient donor T cells. Finally, in analogy to our murine studies, colonic *RORC* expression levels inversely correlated with the presence of GvHD in allo-HSCT patients. Together, this study provides a crucial example of a BATF-dependent, however, IL-23R signaling- and RORγt-, i.e., Th17 fate-independent regulation of a colitogenic T cell population critically impacting the current understanding of intestinal GvHD.

## Introduction

Despite exerting crucial barrier-protective functions under homeostatic conditions, interleukin-17a (IL-17a) producing T helper (Th17) cells are the most-abundant subpopulation within intestinal inflammatory lesions found in patients with inflammatory bowel diseases (IBDs) and acute manifestation of gastrointestinal graft-versus-host disease (GvHD) after allogeneic hematopoietic stem cell transplantation (allo-HSCT) (1, 2). Hence, targeting Th17 cells appears to represent a master-switch to turn off unwanted inflammation in the gut (3–6). Indeed, we and others have previously demonstrated that T cells with a genetic inactivation of Th17 cell regulating transcription factors retinoic acid-related orphan receptor gamma t (RORγt), interferon regulatory factor-4, signal transducer and activator of transcription 3 (STAT3), or basic leucine zipper transcription factor ATF-like (BATF) uniformly failed to induce colitis in a series of syngeneic murine IBD models supporting the concept that harnessing unleashed intestinal inflammation can be achieved by targeting Th17 cell biology (7–11). In line within this assumption, genetic inactivation or blockage of interleukin-23 (IL-23) knowingly absolutely required for the occurrence of Th17 cells *in vivo* were shown to mitigate colitis in preclinical model systems and be effective in treating IBD (5, 12, 13). Overall, these data suggest that IL-23-driven T-cell responses are critically contributing to the manifestation of intestinal inflammation both in murine syngeneic colitis models and in human IBD and hence Th17-centered concepts are highly promising to provide progress for the therapy of IBD in the future.

However, in particular in respect to intestinal GvHD following allo-HSCT, the issue of the selective pathogenic contribution of bona fide Th17 cells to the manifestation of mucosal inflammation has continued to remain essentially unresolved in the light of a series of reports with inconclusive and in part diametrically opposed outcomes resulting in various interpretations of its role by the scientific community (14–16). Interestingly, we recently described that donor T cells lacking the expression of the Th17 lineage regulating transcription factor BATF indeed conferred protection against GvHD-associated colitis both in a major and minor histocompatibility mismatched model of allo-HSCT in mice (17). Importantly, besides the known role in Th17 cell differentiation (18), we found the development of interleukin-7 receptor (IL-7R)-responsive, granulocyte-macrophage colony-stimulating factor (GM-CSF) expressing donor T cells, also termed Th_GM_ cells (19–21), to be hampered in the absence of BATF in these model systems. More importantly, selective blockade of IL-7R^hi^ GM-CSF^+^ T cells alone largely recapitulated the protection that we observed upon the transplantation of BATF-deficient donor lymphocytes (17). Given the dual role of BATF in regulating both Th17 cells and GM-CSF^+^ T cells, these data urged us to further study a number of issues raised by these findings with the goal to ultimately disclose the functional relevance of Th17 cells compared to GM-CSF-expressing T cells in gastrointestinal GvHD. In the light of the notion provided by recent studies showing in experimental autoimmune encephalomyelitis, a murine model of multiple sclerosis, that GM-CSF-expressing T cells are driven by IL-23, express the master regulator of Th17 development RORγt and hence putatively represent a Th17 cell subset (22, 23), our current study was intended to characterize (1) the developmental relationship between Th17 and GM-CSF^+^ T cells based on the dependency on upstream and transcriptional signals and (2) the subset-specific, functional contribution to the manifestation of acute GvHD-associated colitis *in vivo*.

## Materials and Methods

### Mice

Female BALB/cJRj, and C57Bl/6 mice were purchased from Janvier Labs, and congenic CD45.1/Ly5.1 B6.SJL-PtrprcaPepcb/BoyCrl mice were purchased from Charles River Laboratories. 129S-*Batf^tm1.1Kmm^*/J (termed *Batf*^−/−^ mice) and B6.129P2(Cg)-*Rorc^tm2Litt^*/J (termed *Rorc*^−/−^ mice) were purchased from the Jackson Laboratory and intercrossed to generate *Rorc*^−/−^ *Batf*^−/−^ mice. *Il23r*^−/−^ mice [B6NTac;B6N-A<tm1Brd> Il23r<tm2a(EUCOMM)Wtsi/Wtsi] were provided by the Trust Sanger Institute Mouse Genetics Project (Sanger MGP) (24). Mice were maintained under specific pathogen-free conditions. Mice older than 7 weeks were used. This study was carried out in accordance with the recommendations of the government of Mittelfranken in Bavaria, Germany. The protocol was approved by the government of Mittelfranken in Bavaria, Germany.

### Allogeneic Bone Marrow (BM) Transplantation and GvHD Scoring

Graft-versus-host disease was induced and scored as described before (17). Briefly, 10- to 12-week-old female H-2d^+^ Balb/c recipient mice received a single dose of 8 Gy X-ray *via* total body irradiation (day 0). At day 1 after irradiation, BM cells of allogeneic CD45.1/Ly5.1 B6.SJL-*Ptprc^a^ Pepc^b^*/BoyCrl mice were isolated and T cells of the BM were depleted by magnetic bead separation using anti-CD90.2 microbeads (Miltenyi Biotec) according to the manufacturer’s instructions routinely resulting in a depletion of more than 98% of the pre-existing T cell fraction as confirmed by flow cytometry. Then, 5 × 10^6^ T cell depleted BM cells were i.v. injected into recipient mice. At day 2 after irradiation, mice received i.v. 0.7 × 10^6^ allo-reactive CD3^+^ T cells that were isolated from total splenocytes of C57Bl/6 wild type (termed WT mice), *Rorc*^−/−^, *Il23r*^−/−^ and *Rorc*^−/−^ *Batf*^−/−^ mice, respectively, by magnetic separation using the Mouse Pan T cell isolation kit II (Miltenyi Biotec) according to the manufacturer’s instructions. Mice were assessed 3×/week for the presence of clinical GvHD symptoms. Indicated GvHD-associated symptoms (body weight, posture, activity, skin and fur texture, and stool consistency) were individually scored from 0 (no manifestation) to 2 (severe manifestation) and the sum of all individual scores per mouse added up to the clinical GvHD score.

### Mouse Colonoscopy

Macroscopic mucosal inflammation was assessed by colonoscopy using an image 1™ S3 mini-endoscope (Karl Storz) as previously described (25). Therefore, mice were anesthetized by inhalation and inflammation of the colon was estimated using a modified murine endoscopic index of colitis severity based on the following parameters: thickening of the colon bowel wall, changes of the vascularity, granularity of the mucosal surface, and stool consistency. Every parameter was scored from 0 for no colitis to 3 for massive inflammation adding up to a maximum score of 12 as previously described (26).

### *In Vivo* Antibody Treatment of Mice

In studies with *in vivo* antibody treatment, mice received 3×/week 300 µg anti-mouse IL-7R antibody (clone A7R34) starting on the day of BM transplantation (day 1) until day 15 and 300 µg anti-mouse GM-CSF antibody (MP1-22E9) throughout the experiment by i.p. injection. As a control, a group of mice was treated with 300 µg isotype rat IgG2a antibody (clone 2A3) 3×/week over the entire course of the experiment also by i.p. injection. All antibodies were purchased from BioXcell.

### Histopathological Analysis

After euthanizing allo-HSCT recipient mice, the distal segment of the colon was removed, flushed with PBS, and fixed in 4.5% formaldehyde overnight. Sections (3 µm) of paraffin-embedded colon tissue were stained with hematoxylin and eosin and used for histopathological analysis. Inflammation was scored by a to the experimental groups blinded pathologist. GvHD-associated inflammation was scored semiquantitatively as previously described (27): 0 (none or minimal signs); 1 (mild signs); 2 (moderate signs); or 3 (severe signs) (17).

### Colonic Lamina Propria (LP) Cell Isolation

Colonic LP cells were isolated as described before (17). Briefly, the colon was removed from euthanized mice, flushed with PBS to remove intestinal content, and cut into small pieces. After washing colonic pieces twice with HBSS supplemented with 1% EDTA (0.5 mM), intestinal tissue was digested with a solution containing DNase I (0.5 mg/ml), Collagenase D (1 mg/ml), Dispase II (6 U/ml) (all enzymes purchased by Roche), and 5% FCS. Digested tissue was filtered and washed with PBS. LP cells were enriched by density gradient centrifugation where 80% Easycoll was overlaid with cells resuspended in 40% Easycoll (Biochrom). Following centrifugation cells were washed with RPMI supplemented with 10% FCS and stored on ice until analysis.

### Flow Cytometry

For the analysis of cell surface markers, LP cells were stained with fluorochrome-conjugated antibodies for 20 min at 4°C in the dark. After washing with FACS buffer (3% FCS in PBS), cells were analyzed on a FACSFortessa™ II (BD Biosciences) flow cytometer. For intracellular cytokine staining, isolated LP cells (1 × 10^6^/ml) were cultured in supplemented DMEM (containing 10% FCS, 1% penicillin–streptomycin, 1% non-essential amino acids, 1% l-glutamine, and 0.1% β-mercaptoethanol) alone or in the presence of 50 ng/ml phorbol 12-myristate 13-acetate and 1 µM ionomycin for 4 h at 37°C. 1 µg/ml brefeldin A was added for the last 3 h of culture (all purchased from Sigma Aldrich). Thereafter, cells were stained for surface markers. Intracellular cytokine staining was performed as described previously (18). In brief, cells were fixed with 2% paraformaldehyde in PBS for 15 min at room temperature. After washing with FACS buffer, cells were permeabilized with 0.05% saponin (Sigma Aldrich, Germany) in FACS buffer followed by intracellular cytokine staining for 30 min at 4°C in the dark using fluorochrome-labeled antibodies dissolved 0.5% saponin in FACS buffer. After washing cells with 0.05% saponin in FACS buffer, cells were resuspended in FACS buffer cells and analyzed on a FACSFortessa™ II (BD Biosciences) flow cytometer. Data were analyzed using FlowJo 7.6.5 and 10.2 software (Tree Star Inc.). The following antibodies were used: α-CD3ε (17A2, BioLegend), α-CD4 (GK1.5, BioLegend), α-CD8a (53-6.7, BioLegend), α-CD45.1 (A20, BioLegend), α-CD45.2 (104, BioLegend), α-GM-CSF (MP1-22E9, BioLegend), α-IL-17a (TC11-18H10.1, BioLegend), and α-IFNγ (XMG1.2, BioLegend).

### *In Vitro* T Cell Culture

For *in vitro* CD4^+^ T cell cultures, splenic CD4^+^ T cells were isolated by negative selection using magnetic microbeads of the CD4^+^ T Cell Isolation Kit II (Miltenyi Biotec) according to the manufacturer’s instructions. In the case of Th_GM_ cultures, enriched CD4^+^CD25^−^ T cells were further sort-purified using a FACS Aria cell sorter (Becton Dickinson) at the Cell Sorting and Immunomonitoring Core Unit of the University Hospital Erlangen, Friedrich Alexander University Erlangen-Nuremberg. T Cells (1 × 10^6^/ml) were cultured at 37°C in a 24-well-plate in supplemented RPMI (containing 10% FCS, 1% penicillin–streptomycin, 1% non-essential amino acids, 1% l-glutamine, and 0.1% β-mercaptoethanol; Sigma Aldrich) for Th_GM_ differentiation (17) or in serum-free X-VIVO 15 medium (Lonza) supplemented with 1% penicillin–streptomycin for Th17 differentiation (26). T cells were stimulated with 10 µg/ml plate-bound anti-CD3 antibody (clone 145-2C11; BioXcell) and 1 µg/ml soluble anti-CD28 antibody (clone 37.51; BioXcell). For T cell differentiation, T cells were cultured in the presence of 10 µg/ml soluble anti-IFNγ (clone XMG1.2; BioXcell) alone (drift condition) or for Th_GM_ polarizing conditions together with 2 ng/ml recombinant IL-7 (R&D Systems) while for Th17 polarizing conditions 20 ng/ml recombinant IL-1β (Peprotech), 20 ng/ml recombinant IL-6 (Peprotech), and 50 ng/ml recombinant IL-23 (Milenyi Biotec) were added to the cultures. Th_GM_ polarized T cells were harvested at day 3 while Th17 differentiated T cells underwent RNA isolation after 5 days of culture.

### Quantitative Real-Time PCR (qPCR)

RNA was isolated from whole colon tissue using the NucleoSpin™ RNA isolation kit (Macherey Nagel). RNA of *in vitro* differentiated T cells was isolated using the RNeasy Micro Kit (Qiagen). 1 µg of RNA was reversed transcribed into cDNA using iScript™ cDNA Synthesis Kit (Bio-Rad) according to manufacturer’s instructions. qPCR reactions were performed on a CFX Connect and CFX96 Real-Time PCR detection system (Bio-Rad) using iQ™ SYBR^®^ Green Supermix (Bio-Rad). Data were analyzed with CFX Manager v3.1 (Bio-Rad). Expression levels of target genes for each sample were normalized relative to the housekeeping gene HPRT. Relative gene expression levels were calculated with the ΔΔCt method. For the analysis of gene expression within murine colon tissue samples, gene expression levels detected in no T cells (noT) controls were arbitrarily set to 1 and all other gene expression levels were calculated and displayed in relation to the normalized noT controls.

Following primers were used synthesized by MWG Eurofines (Germany): *Hprt* forward 5′-TGG ATA CAG GCC AGA CTT TGT T-3′, reverse 5′-CAG ATT CAA CTT GCG CTC ATC-3′, *Ifng* forward 5′-ATC TGG AGG AAC TGG CAA AA-3′, reverse 5′-TGA GCT CAT TGA ATG CTT GG-3′, *Csf2* forward 5′-ATC AAA GAA GCC CTG AAC CT-3′, reverse 5′-GTG TTT CAC AGT CCG TTT CC-3′, *Tnfa* forward 5′-CTT GTG GCA GGG GCC ACC AC-3′, reverse 5′-CCA TGC CGT TGG CCA GGA GG-3′, *Il-17a* forward 5′-GCT CCA GAA GGC CCT CAG A-3′, reverse 5′-AGC TTT CCC TCC GCA TTG A-3′, *Il1b* forward 5′-GTG ACG TTC CCA TTA GAC AA-3′, reverse 5′-TAT TTT GTC GTT GCT TGG TT-3′, *Rorc* forward 5′-CCG CTG AGA GGG CTT CAC-3′, reverse 5′-TGC AGG AGT AGG CCA CAT TAC A-3′, *Il23r* forward 5′-CAC AAC AAC TAC ACG TCC AT-3′, reverse 5′-TAC CAG TTT CTT GAC ATC GC-3′, *Batf* forward 5′-GGA AGA TTA GAA CCA TGC CTC-3′, reverse 5′-CCA GGT GAA GGG TGT CGG-3′.

### Human Studies

This study was carried out in accordance with the recommendations of the ethics committee of the University Hospital Regensburg. The protocol was approved by the ethics committee of the University Hospital Regensburg. All subjects gave written informed consent in accordance with the Declaration of Helsinki. Colon tissue biopsies of allo-HSCT patients (*n* = 52) were collected during colonoscopy at the Department of Hematology & Oncology and Gastroenterology at the University Hospital Regensburg. Total RNA of human tissue was isolated with the RNeasy Mini Kit (Qiagen) followed by cDNA synthesis using moloney murine leukemia virus reverse transcriptase (Promega) according to the manufacturer’s instructions. Gene expression of RORC and 18S ribosomal RNA was analyzed by quantitative PCR reactions that were performed on a Mastercyler Ep Realplex (Eppendorf) using a QuantiFast SYBR Green PCR Kit (Qiagen). The following primer sets were used: *RORc* forward, 5′-GCA GCG CTC CAA CAT CTT CTC-3′, reverse, 5′-GCA CAC CGT TCC CAC ATC TC-3′; *18SrRNA* forward, 5′-ACC GAT TGG ATG GTT TAG TGA G-3′, reverse, 5′-CCT ACG GAA ACC TTG TTA CGA C-3′. Expression of *RORC* was normalized to *18SrRNA*. The expression levels represent relative units and were calculated from a standard curve plotting three different concentrations of log dilutions against the PCR cycle number as previously described (28). Concurrently colonic tissue sections of the same patients were analyzed for the presence or absence of histopathological signs of intestinal GvHD by a pathologist experienced in GvHD-associated histopathology of the gut during clinical routine. Histopathological scoring of the colitis activity and the presence of GvHD-associated apoptotic events was performed as previously published (29). Apoptotic cells per high power field (hpf) were counted and apoptosis was semiquantitatively scored by two experienced pathologists: 0–1 apoptotic cell/hpf = 0; 2–4 apoptotic cell/hpf = 1; 5–7 apoptotic cell/hpf = 2; >7 apoptotic cell/hpf = 3. Samples with a sore of at least 1 were considered to be positive for apoptosis.

### Statistical Analysis

Unpaired two-tailed Student’s *t*-test was used for comparison of means of two datasets. Clinical GvHD scores were analyzed by two-way ANOVA and Bonferroni posttest. For comparison of gene expression data or colonoscopy scores with more than two datasets, one-way ANOVA and Bonferroni posttest was used. *p < 0.05, **p < 0.01, ***p < 0.001, and ****p < 0.0001 was considered to be significant. Statistical analysis was performed with Graphpad Prism 5 software.

## Results

### IL-23 Receptor Expression on Donor T Cells Is Dispensable for the Manifestation of GvHD-Associated Colitis

**Figure 1:**
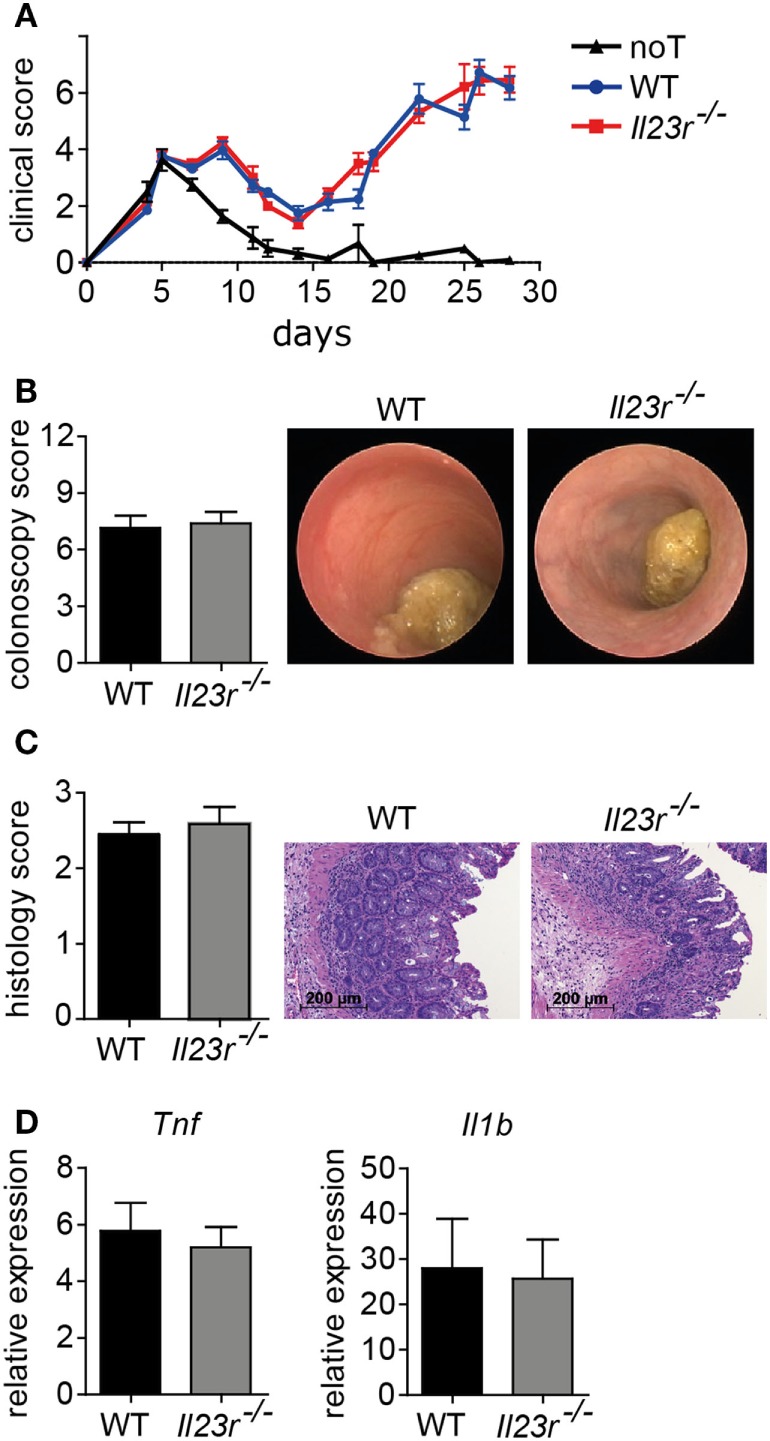
Interleukin-23 receptor (IL-23R) expression by donor T cells is not required for acute graft-versus-host disease (GvHD)-associated colitis manifestation. To induce GvHD, BALB/c mice were irradiated (day 0), received intravenously T cell-depleted bone marrow (BM) (day 1) and were subsequently injected with allogeneic splenic CD3^+^ T cells of C57BL/6 [wild type (WT); *n* = 14] or *Il23r*^−/−^ (*n* = 15) mice (day 2). As a control, mice received T cell-depleted BM alone (noT; *n* = 8). **(A)** Mice were observed three times a week and clinical symptoms of GvHD were scored. Data were analyzed by two-way ANOVA followed by Bonferroni’s multiple comparisons posttest. **(B)** At day 28, colitis severity was assessed by colonoscopy and representative endoscopic images are shown. Mice were sacrificed between day 28 and day 30. **(C)** Histopathological scoring of the inflammation present in the distal colon (WT *n* = 11; *Il23r*^−/−^ *n* = 12). One representative hematoxylin and eosin-stained histopathological cross-section of the distal colon per group is shown. Scale bars: 200 µm. **(D)** Transcript levels of *Tnf* and *Il1b* in colonic tissue of WT (*n* = 14) or *Il23r*^−/−^ (*n* = 15) T cell receiving mice as described above were analyzed by quantitative real-time PCR. Data are combined from two individual experiments. Data were analyzed by Student’s *t*-test and are shown as mean ± SEM.

Previously, we reported that acute inflammatory tissue manifestation of GvHD in the lower gastrointestinal tract depends on the ability to express BATF within donor T cells (17). Surprisingly, BATF-deficient T cells were severely compromised in their ability to differentiate into IL-7R responsive GM-CSF-expressing T cells. Prompted by this finding, we assessed the functional contribution of these T cells and found that indeed combined blockade of IL-7R/IL-7 interaction and GM-CSF proved to be very effective in harnessing GvHD driven colonic inflammation. However, given the dichotomic role of BATF in governing both the differentiation of IL-17-producing Th17 cells and GM-CSF-producing T cells and the published concept on the IL-23 dependency of GM-CSF expressing Th17 cells as crucial drivers of tissue inflammation, e.g., in the central nervous system (17, 18, 22, 23), we reasoned that additional studies are required to clarify the precise, subset-specific functional contribution of bona fide Th17 cells to the manifestation of acute GvHD-associated colitis upon allo-HSCT. To achieve this goal, we first sought to assess whether the ability of the donor T cell to directly respond to IL-23 *via* IL-23/IL-23 receptor (IL-23R)-interaction on donor T cells is functionally relevant for the manifestation of intestinal GvHD. Importantly, employing a full MHC class I mismatch model, we failed to observe any protective effects on the clinical course of GvHD upon transplantation of IL-23R-deficient compared to WT CD3^+^ donor lymphocytes (Figure 1A). Moreover, intestinal GvHD as assessed by both endoscopic and histopathological evaluations of matching colonic tissue specimen was indistinguishably detectable in mice receiving either WT or IL-23R-deficient donor T cells (Figures 1B,C). Also on a molecular level, gene expression profiling analyses of proinflammatory cytokines known to promote inflammatory tissue damage in the colon demonstrated that *Il1b* and *Tnf* levels are comparably upregulated independent of the T cell’s ability to express IL-23R (Figure 1D). Overall, these data demonstrate that direct effects of IL-23 *via* IL-23R expression on the intestinal GvHD-mediating donor T cell population are largely dispensable for disease manifestation.

### Donor T Cell-Intrinsic RORγt Expression Does Not Drive Acute Colonic Inflammation Upon Allo-HSCT

**Figure 2:**
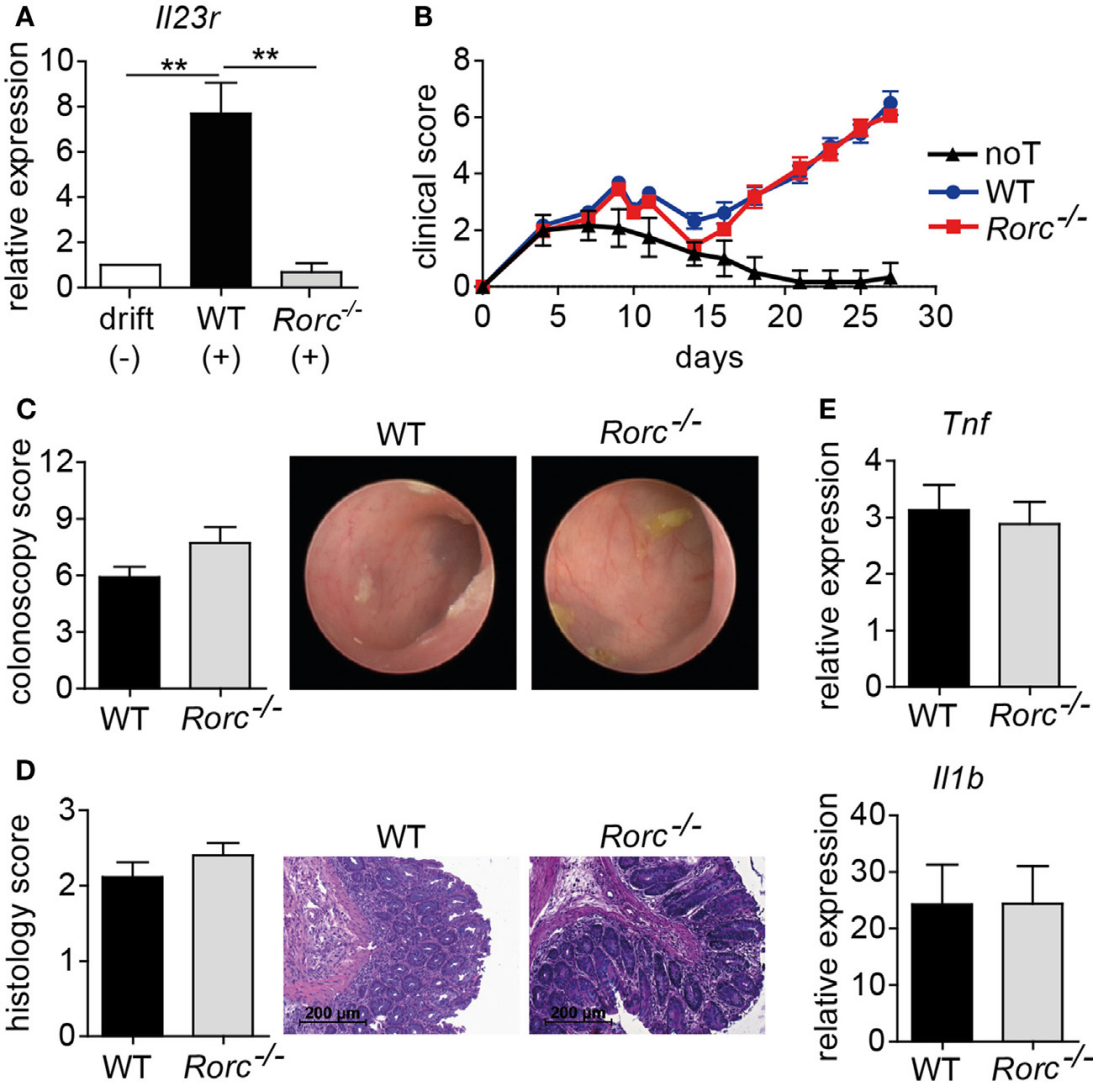
Retinoic acid-related orphan receptor gamma t (RORγt)-dependent T helper (Th17) cells are dispensable for acute intestinal graft-versus-host disease (GvHD). **(A)** Naïve CD4^+^ T cells were isolated from the spleen by negative selection using magnetic microbeads and *in vitro* polarized into inflammatory Th17 cells by co-culturing anti-CD3/anti-CD28 activated T cells in the presence of anti-IFNγ antibodies and either without (−) or with (+) recombinant interleukin (IL)-1β, IL-6, and IL-23. After 5 days, T cells were harvested, RNA was isolated, and transcribed into cDNA followed by quantitative real-time PCR (qPCR) analyses of *Il23r* transcript levels. Gene expression levels detected within T cells cultured under drift conditions were arbitrarily set down to 1 and all other gene expression levels were normalized to the expression level of this control. Data are combined from two individual experiments and were analyzed by one-way ANOVA followed by Bonferroni’s multiple comparisons posttest. **p < 0.01 was considered significant. Data are shown as mean ± SEM. **(B–E)** For GvHD induction, BALB/c mice were irradiated (day 0), transplanted with T cell-depleted bone marrow (BM) (day 1) and subsequently injected with allogeneic splenic CD3^+^ T cells of wild-type (WT) (*n* = 18) or *Rorc*^−/−^ (*n* = 23) mice (day 2). As a control, one group of mice received T cell-depleted BM alone [no T cells (noT); *n* = 6]. **(B)** Clinical symptoms of GvHD mice were scored three times a week. Pooled data from three independent experiments are shown and were analyzed by two-way ANOVA followed by Bonferroni’s multiple comparisons posttest. **(C)** Between day 28 and day 30, colonic inflammation of WT (*n* = 12) or *Rorc*^−/−^ (*n* = 11) T cell receiving mice was assessed by colonoscopy and representative endoscopic images are shown. Mice were sacrificed the day after colonoscopy. **(D)** Histopathological scoring of the colitis activity within the distal colon of WT (*n* = 9) or *Rorc*^−/−^ (*n* = 10) T cell receiving mice was performed. One representative hematoxylin and eosin-stained histological cross-section of the distal colon per group is shown. Scale bars: 200 µm. Shown results represent pooled data from two individual experiments. **(E)** Gene expression levels detected within colonic tissue of WT or *Rorc*^−/−^ T cell receiving mice of *Tnf* (WT *n* = 16; *Rorc*^−/−^ *n* = 12) and *Il1b* (WT *n* = 17; *Rorc*^−/−^ *n* = 14) around day 30 were analyzed by qPCR. Data display pooled data from at least three individual experiments. Data were analyzed by Student’s *t*-test and are shown as mean ± SEM.

Interleukin-1β/IL-6/IL-23-induced inflammatory Th17 cells consecutively upregulate IL-23R expression which renders these T cells progressively responsive to IL-23, thereby further promoting this differentiation pathway (Figure 2A) (30). Importantly, we and others have demonstrated that the induction of IL-23R expression is regulated by and dependent on bona fide Th17 cell differentiation-regulating transcription factors as, e.g., BATF and RORγt (Figure 2A) (26, 30). However, IL-23 responsiveness due to IL-23R expression is only one facet of Th17 cells (3). Therefore, we assessed whether RORγt-deficient T cells conferred protection against intestinal GvHD manifestation given the fact that RORγt represents a Th17 defining transcription factor that is highly related to BATF. More importantly, under syngeneic conditions, RORγt- and BATF-deficient T cells comparably failed to induce colitis upon transfer into lymphopenic *Rag1*^−/−^ mice (9, 11). Strikingly, however, and in clear delineation from the protection conferred by BATF-deficient T cells, upon allo-HSCT WT and RORγt-deficient T cells induced virtually identical signs of systemic GvHD. Moreover, endoscopic assessment of GvHD-associated colitis revealed indistinguishable intestinal GvHD phenotypes between both groups of donor T cell receiving mice (Figures 2B,C). Histopathological evaluation of matching tissue specimen confirmed that colonic inflammation was comparably detectable in both groups (Figure 2D). Similar to our results obtained in the studies on the role of IL-23R expression on T cells, proinflammatory cytokine gene expression analyses (*Il1b* and *Tnf*) within GvHD-affected colon tissues samples yielded similar results between mice transplanted with WT and RORγt-deficient donor T cells (Figure 2E). Together, these series of studies demonstrate that both IL-23R and RORγt expression within the donor T cell fraction are seemingly dispensable for intestinal GvHD manifestation.

### Intestinal GvHD Develops Despite Hampered Th17 Differentiation due to RORγt-Independent Th1 and GM-CSF-Expressing T Cell Formation

**Figure 3:**
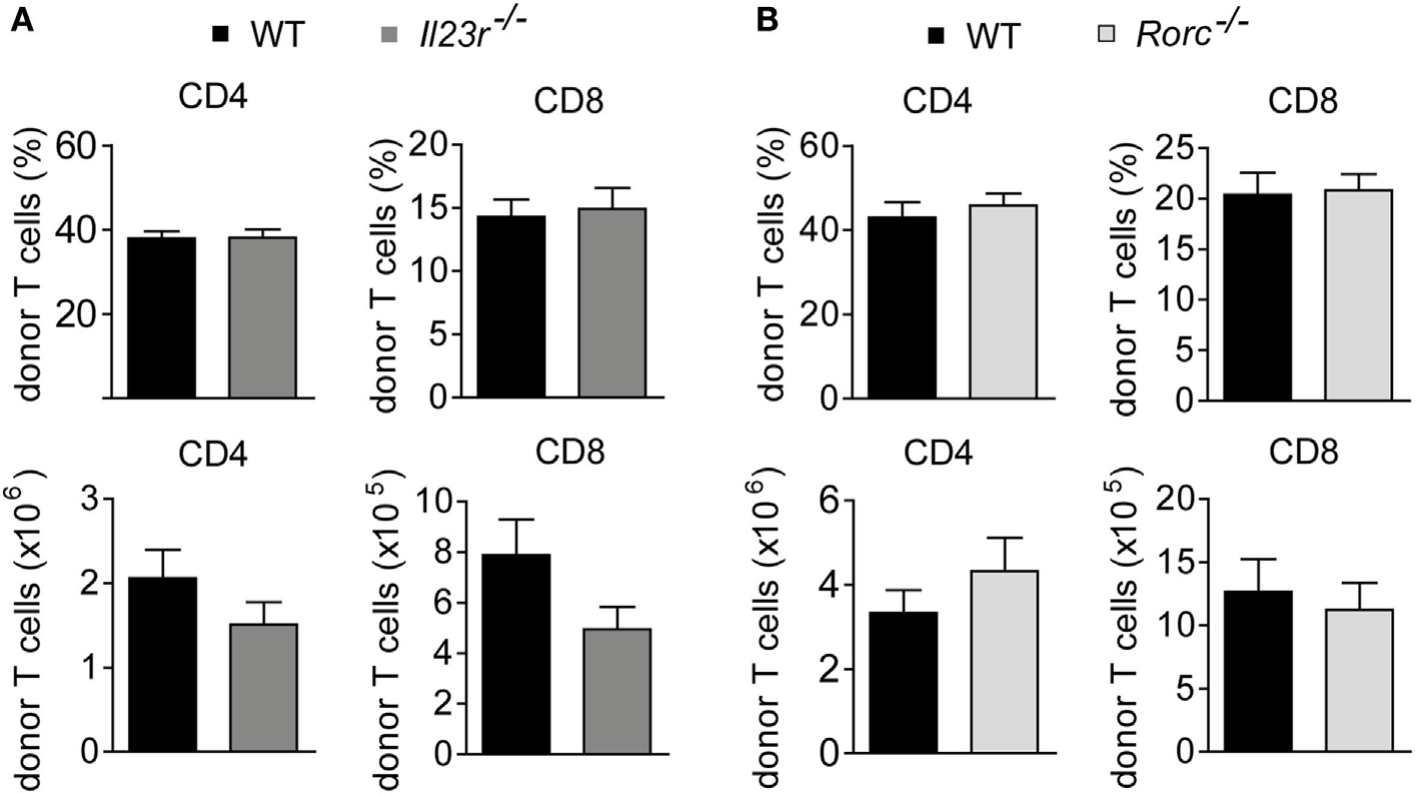
Colonic donor T cell expansion occurs in an interleukin-23 receptor (IL-23R)- and retinoic acid-related orphan receptor gamma t-independent manner. At day 28–31, colonic lamina propria mononuclear cells of allogeneic hematopoietic stem cell transplantation BALB/c mice with fully established clinical signs of intestinal graft-versus-host disease due to the transfer of allogeneic T cells derived from wild-type (WT) (*n* = 14) and *Il23r*^−/−^ (*n* = 15) mice **(A)** or WT (*n* = 12) and *Rorc*^−/−^ (*n* = 11) mice **(B)** were analyzed by flow cytometry. The frequency and absolute number of CD4^+^ and CD8^+^ donor T cells are displayed. Data represent pooled results from two independent experiments and were analyzed by Student’s *t*-test. Data display mean ± SEM.

**Figure 4:**
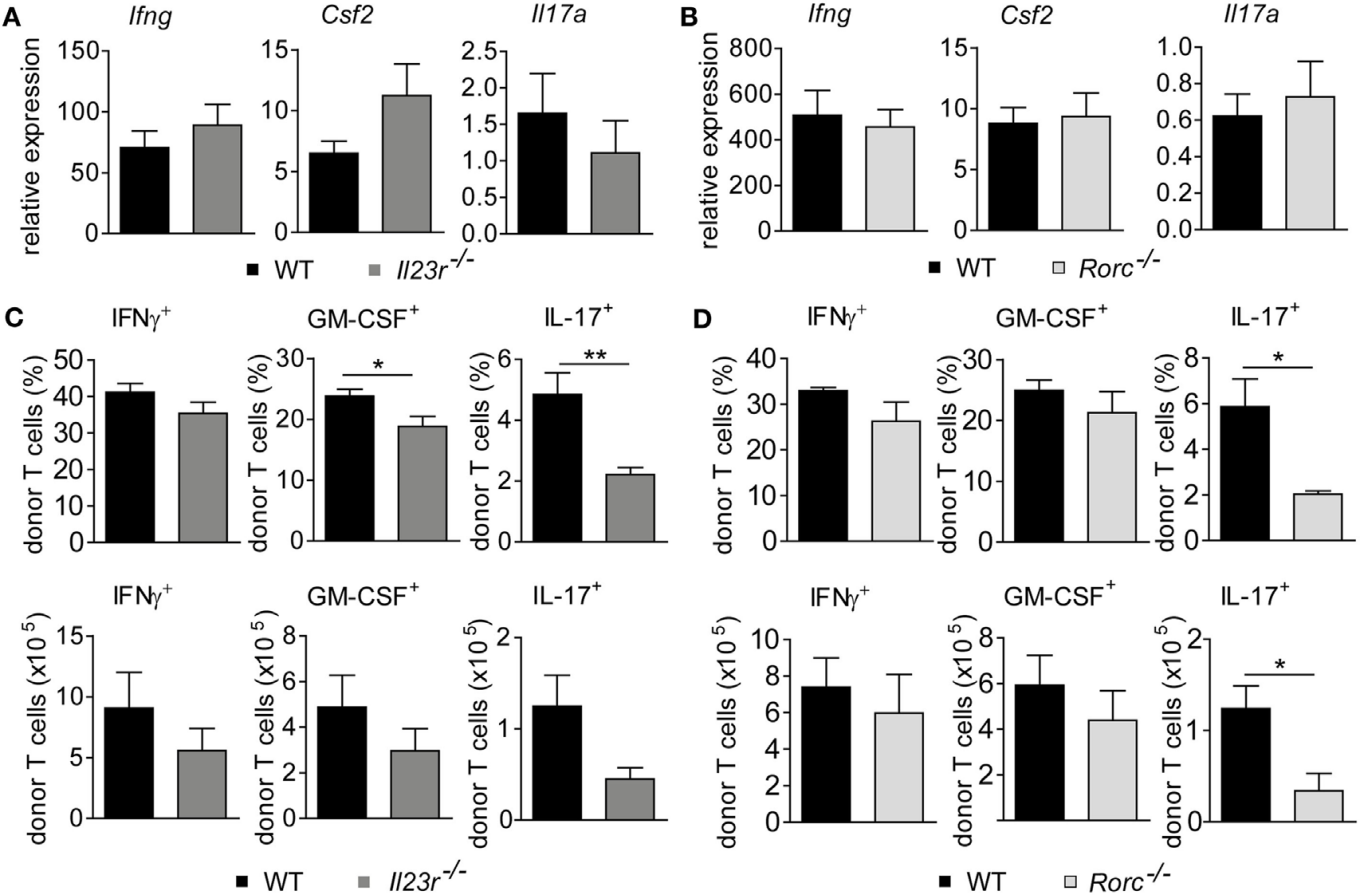
Genetic depletion of retinoic acid-related orphan receptor gamma t-dependent T helper cells preserves the granulocyte-macrophage colony-stimulating factor (GM-CSF) expressing donor T cell pool. Experimental graft-versus-host disease (GvHD) induction was performed as described in Figures 1 and 2. **(A)** Gene expression levels in total colonic tissue of wild-type (WT) and *Il23r*^−/−^ donor T cell receiving mice of *Ifng, Csf2* (WT *n* = 14; *Il23r*^−/−^ *n* = 15) and *Il17a* (WT *n* = 9; *Il23r*^−/−^ *n* = 5) transcripts were analyzed by quantitative real-time PCR (qPCR). **(B)** Gene expression levels in total colonic tissue of WT and *Rorc*^−/−^ donor T cell receiving mice of *Ifng, Csf2* (WT *n* = 17; *Rorc*^−/−^ *n* = 14) and *Il17a* (WT *n* = 7; *Rorc*^−/−^ *n* = 8) transcripts were analyzed and quantitated by qPCR around day 30, i.e., when allogeneic hematopoietic stem cell transplantation mice displayed fully established signs of intestinal GvHD. Data represent pooled data from two **(A)** and at least three **(B)** individual experiments, respectively. **(C,D)** In addition, lamina propria mononuclear cells of WT (*n* = 6) and *Il23r*^−/−^ (*n* = 6) T cell receiving mice **(C)** or WT (*n* = 4) and *Rorc*^−/−^ (*n* = 3) T cell receiving mice **(D)** were assessed by flow cytometry for the frequencies and absolute numbers of GM-CSF-, IFNγ- or interleukin 17a (IL-17a)-producing CD4^+^ donor T cells, respectively, employing intracellular cytokine staining techniques. Displayed data are derived from one representative of two independent experiments and were analyzed by Student’s *t*-test. *p < 0.05 and **p < 0.01 was considered significant. All data are shown as mean ± SEM.

Further studies showed that both frequencies and absolute numbers of colonic LP-infiltrating CD4^+^ and CD8^+^ donor T cells were indistinguishable between WT and either IL-23R-deficient or RORγt-deficient donor T cells (Figures 3A,B). Hence, given the virtually identical intestinal GvHD phenotypes, we reasoned that also the quality of colonic GvHD-mediating T cell subsets might be unaffected by IL-23R- and RORγt-, i.e., Th17 deficiency, within the donor T cell compartment. To address this point, we first quantitated preferentially T cell-derived cytokine gene expression levels in total colon tissue samples of GvHD-affected and control mice (Figures 4A,B). Interestingly, *Il17a* is largely regulated in a GvHD-independent manner given our finding that *Il17a* gene expression was barely induced in colon tissues of GvHD-affected mice irrespective of the genotype of the GvHD-inducing donor T cells when compared to mice receiving T cell-depleted BM alone (Figure 4A). In contrast to *Il17a, Ifng*, and *Csf2* gene expression were significantly upregulated in the presence of intestinal GvHD. However, we failed to detect any negative regulatory effect on the expression of proinflammatory cytokine genes resulting from the usage of either IL-23R- or RORγt-deficient compared to WT donor T cells (Figures 4A,B). To further functionally characterize colonic donor-derived CD4^+^ T cell subsets, we performed flow cytometry and intracellular cytokine expression profiling experiments of LP-derived T cells *ex vivo*. Expectedly, these studies confirmed that both donor T cell-restricted genetic inactivation of IL-23R and RORγt, respectively, largely abrogated the ability of donor T cells to differentiate into Th17 cells (Figures 4C,D). By contrast, despite a slightly, however, significantly reduced frequency of GM-CSF-expressing IL-23R-deficient T cells, the absolute numbers of IFNγ-expressing Th1 cells and GM-CSF-expressing LP donor T cells remained unaffected in the absence of either IL-23R or RORγt expression within donor T cells (Figures 4C,D). Interestingly, quantification of indicated donor T cell subsets demonstrated that Th1 and GM-CSF-producing T cells combined absolutely outnumbered IL-17a-producing Th17 cells within the colonic LP compartment of GvHD-affected mice by about 10- to 15-fold. Overall, these data provide substantial experimental evidence for the conclusion that the donor T cells ability to directly respond to IL-23, i.e., express a functional IL-23R, and to express the master regulator of Th17 cell differentiation RORγt is dispensable for the colonic manifestation of intestinal GVHD.

### BATF Deficiency Abrogates RORγt-Independent Donor T Cell-Mediated Colonic GvHD

**Figure 5:**
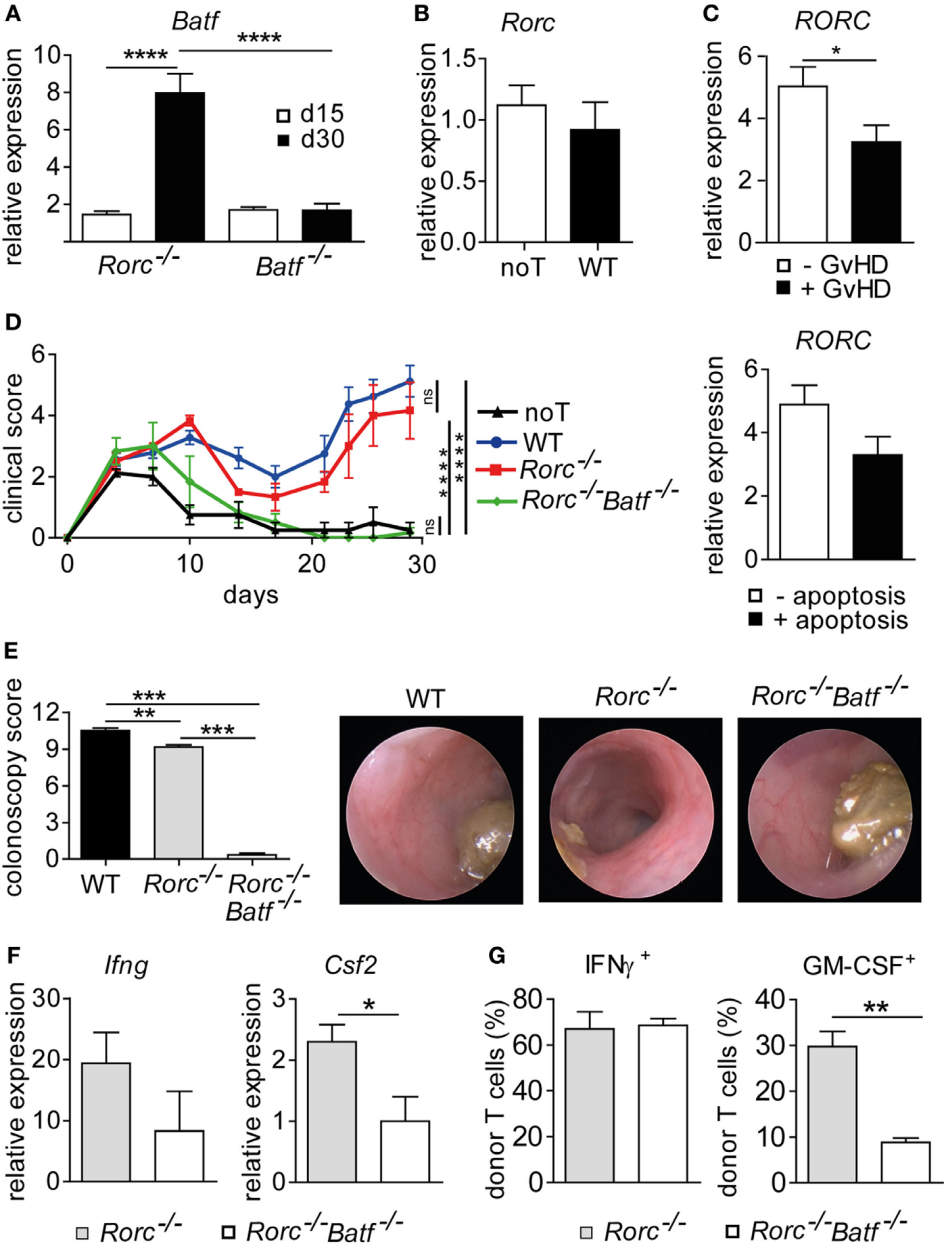
Retinoic acid-related orphan receptor gamma t-independent intestinal graft-versus-host disease (GvHD) is controlled by basic leucine zipper transcription factor ATF-like (BATF). **(A)** Transcript levels of *Batf* in colonic tissue of allo-HSCT BALB/c mice with severe signs of intestinal GvHD following transplantation of allogeneic CD3^+^ T cells derived from of *Batf*^−/−^ and *Rorc*^−/−^ mice were measured by quantitative real-time PCR (qPCR) at day 15 (*Batf*^−/−^ *n* = 12; *Rorc*^−/−^ *n* = 2) and day 30 (*Batf*^−/−^ *n* = 12; *Rorc*^−/−^ *n* = 4). Data represent pooled data from two independent experiments. Gene expression levels detected in colonic tissues of no T cells (noT) controls at day 15 were arbitrarily set down to 1 and all other gene expression levels were normalized to the expression level detected within noT controls. **(B)** Transcript levels of *Rorc* in colonic tissue of irradiated BALB/c mice transplanted with T cell depleted bone marrow alone (noT, *n* = 11) or followed by injection of allogeneic CD3^+^ T cells of wild-type (WT) (*n* = 12) mice were measured by qPCR at day 30. **(C)** Transcript levels of *RORC* in colonic tissue biopsies from allo-HCT patients were measured by qPCR. Samples were grouped into indicated categories based on the absence (−GvHD; *n* = 30) or presence (+GvHD; *n* = 22) of GvHD-associated histopathological lesions or by the absence (−apoptosis; *n* = 32) or presence (+apoptosis; *n* = 20) of GvHD-related epithelial cell apoptosis. Data are shown as normalized relative *RORC* expression levels calculated from a standard curve. **(D–G)** Experimental GvHD induction was achieved as described in Figures 1 and 2. To this end, allogeneic hematopoietic stem cell transplantation BALB/c mice were transplanted with allogeneic CD3^+^ T cells derived from WT (*n* = 4), *Rorc*^−/−^ (*n* = 3), or *Rorc*^−/−^*Batf*^−/−^ (*n* = 3) mice or received no T cells (noT) as a control (*n* = 4). **(D)** Mice were assessed three times a week for signs and severity of systemic GvHD. Data were analyzed by two-way ANOVA testing followed by Bonferroni’s multiple comparisons posttest. ****p < 0.0001 was considered significant, whereas *p* > 0.05 indicates no significance (ns). **(E)** At day 28, colitis severity was determined by colonoscopy and representative endoscopic images are shown. **(F)** Expression levels of *Ifng* and *Csf2* transcripts within colonic tissues of *Rorc*^−/−^ (*n* = 7) or *Rorc*^−/−^*Batf*^−/−^ (*n* = 3) T cell receiving mice were analyzed by qPCR. Data are shown from at least one experiment. **(G)** Colonic lamina propria mononuclear cells of indicated donor T cell receiving mice were isolated and the frequencies of granulocyte-macrophage colony-stimulating factor (GM-CSF^+^) or IFNγ^+^ CD4^+^ donor T cells were assessed by flow cytometry employing intracellular cytokine staining techniques. Data of panels **(A,E)** were analyzed by one-way ANOVA followed by Bonferroni’s multiple comparisons posttest. **p < 0.01, ***p < 0.001, and ****p < 0.0001 was considered significant. Data of panels **(B,C,F,G)** were analyzed by Student’s *t*-test. *p < 0.05 and **p < 0.01 was considered significant. All data are shown as mean ± SEM.

T cells polarized under Th17 conditions upregulate both *Rorc* and *Batf* with the former being shown to occur downstream of *Batf* (18). However, given our findings that acute GvHD-associated colitis depends on donor T cell-intrinsic BATF but not RORγt expression, we sought to further explore how *Batf* expression in the inflamed colon relates to the presence of RORγt- and BATF-expressing donor T cells, respectively. For this, we transferred either RORγt- or BATF-deficient donor T cells and assessed colonic *Batf* expression levels around onset (day 15) and during fully established intestinal GvHD (day 30) (Figures 5A,B). Strikingly, despite abrogated Th17 potential, the transfer of RORγt-deficient T cells resulted in a significant upregulation of colonic *Batf* expression while upregulation of *Batf* was not observed upon transplantation of BATF-deficient donor T cells lacking intestinal GvHD-inducing potential (Figure 5A). Interestingly, we conversely found that *Rorc* expression levels in the colon were comparable between mice displaying severe signs of intestinal GvHD and GvHD-free noT controls due to receiving T cell depleted BM alone (Figure 5B). Importantly, quantification of RORC gene expression within human samples derived from allo-HSCT patients with or without GvHD revealed significantly downregulated colonic *RORC* levels in the presence of GvHD, thereby mirroring results of our murine studies (Figure 5C). These data collectively suggest that elevated *Batf* colon tissue expression levels reflect the presence of BATF-expressing, intestinal GvHD-mediating donor T cells while T cell-intrinsic RORγt expression is completely dispensable in this matter. Therefore, we hypothesized that while RORγt and BATF commonly regulate Th17 cell formation critically affecting, e.g., syngeneic colitis development (9, 11, 18, 31), in intestinal GvHD pathogenesis, however, RORγt is not involved in the formation of a second, previously as BATF-dependent described colitogenic T cell subset. To functionally test whether RORγt-independent manifestation of intestinal GvHD is indeed dependent on BATF, we generated RORγt-deficient T cells additionally lacking BATF by intercrossing BATF- and RORγt-deficient mice. As shown in Figures 5D,E, RORγt/BATF double-deficient donor T cells failed to induce allo-response-driven systemic signs of GvHD and endoscopically assessed manifestations of acute colitis while both systemic and intestinal GvHD phenotypes were again virtually indistinguishable between WT and RORγt-deficient T cells. In line with hampered GvHD induction, gene expression profiling analyses of whole colon tissue samples revealed that RORγt/BATF double-deficiency within the donor T cell compartment resulted in reduced expression of proinflammatory mediators (*Ifng, Csf2*) compared to RORγt-deficient T cell receiving mice displaying severe signs of intestinal GvHD (Figure 5F). Finally, while the pool of Th1-differentiated IFNγ^+^ donor T cells was largely unaltered, GM-CSF-expressing T cells were diminished in the absence of both RORγt and BATF, thereby underscoring the recently described role for BATF in this pathway (Figure 5G). Together, these results indicate that RORγt- and hence Th17-independent manifestation of intestinal GvHD depends on the preserved BATF expression within donor T cells and correlates with its abilities to sufficiently mount a GM-CSF^+^ donor T cell pool *in vivo*.

### Donor RORγt/Th17-Independent Colonic GvHD Is Driven by IL-7/IL-7R Interaction and GM-CSF

**Figure 6:**
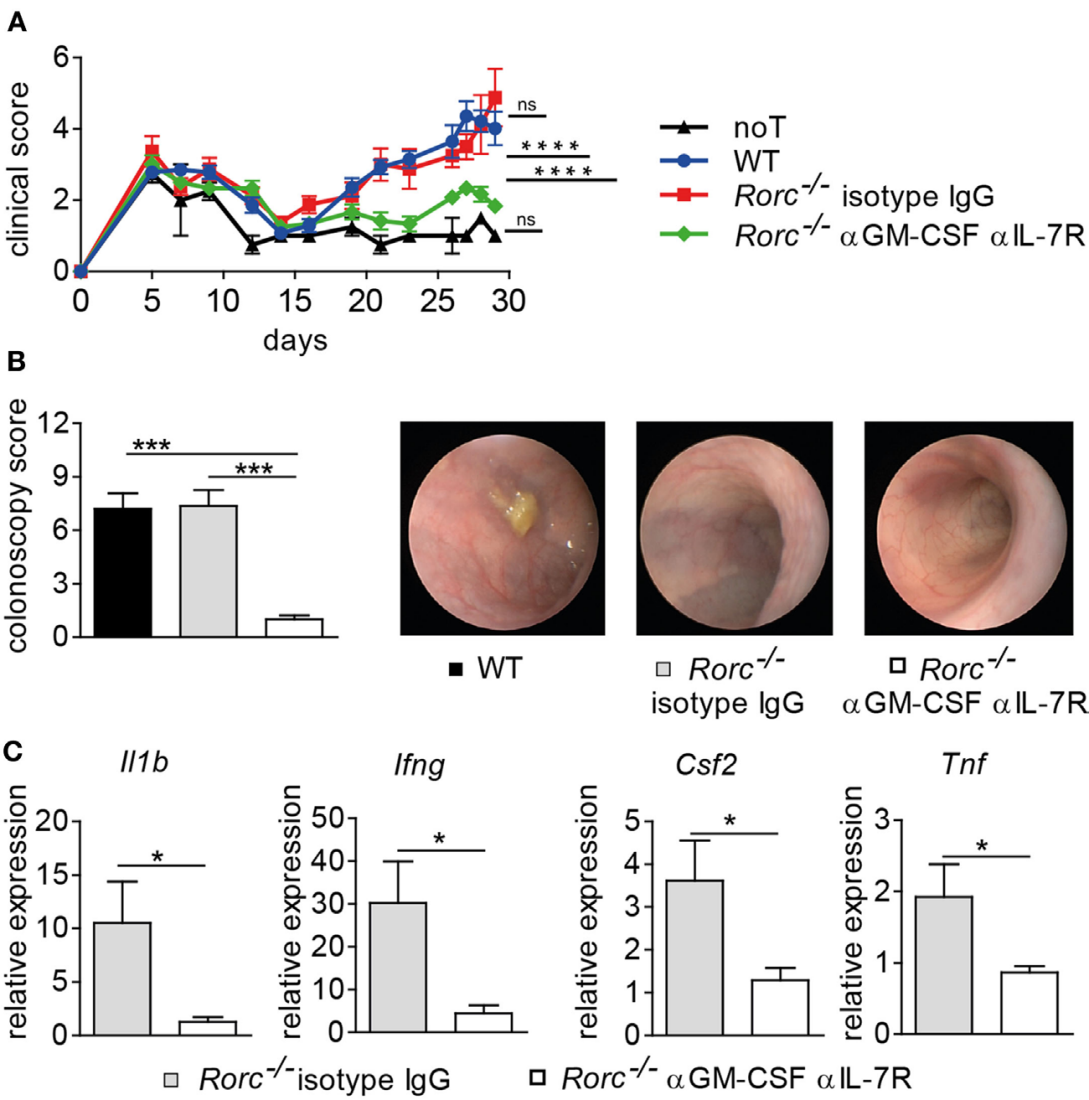
Blockade of interleukin-7 receptor (IL-7R) and granulocyte-macrophage colony-stimulating factor (GM-CSF) mitigates graft-versus-host disease (GvHD)-associated colitis elicited by retinoic acid-related orphan receptor gamma t-deficient donor T cells. Irradiated BALB/c mice received T cell depleted bone marrow alone [no T cells (noT), *n* = 2] or were subsequently injected with allogeneic pan T cells of wild-type (WT) (*n* = 6) or *Rorc*^−/−^ mice. One group of *Rorc*^−/−^ T cell receiving mice was treated 3×/week with anti-GM-CSF antibody (αGM-CSF) throughout the experiment together with anti-IL7R (αIL-7R) until day 15 (*n* = 5) while another group was treated with isotype IgG antibody (*n* = 4) alone. Data are shown from one representative experiment. **(A)** Mice were evaluated 3×/week for the presence of clinical symptoms of GvHD and its severity was accordingly scored. Data were analyzed by two-way ANOVA testing followed by Bonferroni’s multiple comparisons posttest. ****p < 0.0001 was considered significant, whereas *p* > 0.05 indicates no significance (ns). **(B)** At day 29, colitis activity was assessed by colonoscopy and representative endoscopic images are shown. Data were analyzed by two-way ANOVA testing followed by Bonferroni’s multiple comparisons posttest. ***p < 0.001 was considered significant. Mice were sacrificed day 30. **(C)** Transcript levels of *Tnf, Il1b, Ifng*, and *Csf2* in colonic tissue of *Rorc*^−/−^ T cell receiving mice treated with anti-GM-CSF together with anti-IL-7R or with isotype IgG antibody alone were analyzed by quantitative real-time PCR. Gene expression data were analyzed by Student’s *t*-test. *p < 0.05 was considered significant. All data are shown as mean ± SEM.

Our results so far suggest that while BATF and RORγt commonly and sequentially control IL-23R-dependent Th17 cell formation, only BATF but not RORγt positively regulates the GM-CSF-expressing T cell pool recently reported to drive colonic inflammatory manifestations of GvHD. Interestingly, it was recently reported that IL-7 promotes the *in vitro*-formation of GM-CSF-expressing T cells (Th_GM_ cells) due to their dependence on the transcription factor STAT5 (19). In contrast to T cells cultured in the presence of IL-23 and hence under inflammatory Th17 cell-promoting conditions (Figure 2A), we interestingly found that IL-7 supplementation of T cell cultures favoring Th_GM_ polarization did not result in the upregulation of IL-23R expression compared to naïve T cells that were assessed prior culturing (Figure S1 in Supplementary Material). This result that at least *in vitro* differentiated Th_GM_ cells are unresponsive to direct effects exerted by IL-23 substantially extends our previous observation that GM-CSF^+^ T cells develop *in vivo* in an IL-23R-independent manner (Figure 4C). However, our assumption that RORγt-independent formation of intestinal GvHD is mediated by BATF-dependent GM-CSF^+^ T cells is based on results that we previously obtained in studies employing T cells with a selective inactivation of the *Batf* gene (17). However, by taking the approach of co-inactivating two highly potent transcriptional regulators, our results might be confounded by the introduction of additional functional defects in the donor T cell compartment. Hence, we sought to selectively assess the contribution of GM-CSF to the manifestation of RORγt-independent intestinal GvHD by treating RORγt-deficient donor T cell receiving mice either with an IL-7R blocking antibody over the first half of the observation period together and anti-GM-CSF antibody throughout the experiment or with an isotype control antibody alone as described before (17). Strikingly, combined antibody-mediated blockade of IL-7R and GM-CSF but not continuous therapy with the control antibody alone resulted in a significant reduction of clinical signs of systemic GvHD elicited by RORγt-deficient T cells (Figure 6A). Moreover, colonoscopic evaluation of the intestinal GvHD phenotype revealed that RORγt-independent GvHD-associated colitis was mitigated upon dual antibody but not control antibody treatment (Figure 6B). The therapeutic effect on endoscopic signs of colitis was accompanied by diminished expression levels of proinflammatory mediators within colon tissues as we detected reduced amounts of *Il1b, Tnf, Csf2*, and *Ifng* transcripts in anti-IL-7R/anti-GM-CSF- compared to control antibody-treated mice (Figure 6C). Collectively, the results of our current study demonstrate that acute manifestation of intestinal GvHD occurs independent of IL-23R- and RORγt-expressing T cells with the latter being generally accepted to represent bona fide Th17 cells. Mechanistically, GM-CSF^+^ T cells infiltrate the gut, expand in IL-7/IL-7R-interaction dependent, however IL-23R- and RORγt-independent manner and are major mediators of intestinal GvHD observed in the absence of T cell-intrinsic RORγt expression. Together, our study results strongly suggest that selective RORγt- and hence Th17-targeting represent an ineffective approach to limit allo-response driven intestinal inflammation.

## Discussion

Allo-response driven T cell-mediated mucosal inflammation is observed in about 50% of the patients undergoing allo-HSCT mostly because of an otherwise incurable hematopoietic malignancy and routinely represents a clinical challenge due to limited therapeutic options and often therapy-refractory disease courses (32, 33). However, manifestations found in patients suffering from intestinal GvHD share a series of morphological, functional, and immunological characteristics with inflammatory lesions frequently observed in IBD patients (34). In respect to the immunological abnormalities most prominently found in both disease entities, T cells with a Th17 phenotype are similarly overrepresented in patients displaying severe manifestations of acute intestinal GvHD (2). However, while there is both overwhelming preclinical and substantially increasing clinical evidence for a central pathogenetic role of IL-23 and bona fide Th17 cells in promoting intestinal inflammation in IBD (1), pathways dysregulated within donor T cells and hence contributing to the allo-response-driven manifestation of intestinal GvHD following allo-HSCT have been less well deciphered (1, 5, 13, 32, 33, 35, 36). In part due to the discordant usage of various preclinical model systems to induce experimental GvHD, genetic model systems to modulate donor T cell biology and overall non-overlapping experimental setups, it remains difficult to cross-compare results obtained by these studies to draw a clear conclusion (14–16). Furthermore, these studies were generally not designed to definitively discriminate between the functional contribution of bona fide IL-23-driven Th17 cells and of alternatively differentiated, however, Th17-related T cells as described, e.g., in the context of neuro-inflammation with the description of GM-CSF-producing T cells as the major mediator of tissue damage (22, 23, 37).

Recently, we elucidated the central role of BATF-expressing donor T cells in mediating acute intestinal GvHD (17). However, T cell-intrinsic BATF simultaneously controls both *in vitro* and *in vivo* the formation of IL-23-driven Th17 cells and IL-7-driven GM-CSF-expressing T cells, also called Th_GM_ cells (19), thereby excluding the possibility to selectively assess the role of Th17 cells. Hence, we sought to assess the functional contribution of IL-23R expression by donor T cells since IL-23R/IL-23 interaction is critically contributing to Th17 cell formation *in vivo*. Strikingly, employing the same model system and the same conditions under which BATF-deficient T cells—similarly devoid of IL-23R expression due to its BATF-dependent expression—conferred protection IL-23R-deficiency among donor T cells did not confer disease-preventive effects. However, this result essentially conflicts with data from another study that reported reduced mortality and subtle, however, significant reduction of colitis activity in the colon but no other GvHD-affected organs (38). We believe that a series of model-related aspects (e.g., conditioning regimen, composition of the resident intestinal microbiota within the recipient mice in the animal facility, employed genetic mouse models, and most importantly magnitude of the transferred donor T cell pool) may account for the observed differences in the GvHD phenotype best deduced from and exemplified by the parameter “survival”: while in the other study two-thirds of the cohort of WT donor T cell receiving mice succumbed presumably due to rapid and exaggerated manifestation of systemic GvHD within the 30-day-interval after allo-HSCT (38), in our model system the kinetics of both systemic and intestinal GvHD manifestations is purposefully more moderate presumably mostly due to the transfer of a lower T cell dose to induce GvHD. Importantly, however, we reason at that point that our study reflects a presumably clinically more relevant setting since the manifestation of intestinal versus systemic GvHD displays a kinetics-wise more physiological pattern observed in GvHD-affected allo-HSCT patients.

Given the fact that IL-23R expression is only one, however, though both phenotypically and functionally critical hallmark of Th17 cells, we sought to assess whether RORγt-deficient T cells would exert comparably protective effects as previously observed in the absence of T cell-intrinsic BATF expression. RORγt was reported to represent the master regulator of Th17 cell differentiation (31), acts in concert with BATF in this pathway and cell type (18) and its absence provides full protection in syngeneic colitis models in a manner comparable to BATF deficiency (8, 9, 11). Strikingly, however, we failed to detect any GvHD-alleviating effects in regard to both systemic and intestinal inflammation following the transplantation of RORγt-deficient T cells when compared to WT T cell controls. Furthermore, in contrast to BATF (17), we were unable to detect any RORγt upregulation within both murine and human tissues irrespective whether GvHD was present or absent in allo-HSCT mice or men. However, the absence of IL-23R expression as well as RORγt expression within donor T cells expectedly led to a significant reduction of IL-17a-expressing T cells. Moreover, colonic T cells expressing the Th1 hallmark signature cytokine IFNγ were present in an IL-23R- and RORγt-independent manner. Strikingly and in clear delineation from the results obtained when we employed BATF-deficient T cells (17), RORγt-deficient T cells retained the ability to differentiate into allo-reactive GM-CSF-producing T cells. However, both GvHD-inducing capacity and GM-CSF expression of RORγt-deficient T cells were completely dependent on preserved T cell-intrinsic BATF expression since combined ablation of RORγt and BATF abrogated both GvHD manifestation and the GM-CSF expressing T cells. Furthermore, combined antibody-mediated blockade of the IL-7R and GM-CSF completely abrogated signs of systemic and intestinal GvHD elicited by RORγt-deficient T cells. Interestingly, in support of our hypothesis that IL-23R signaling-dependent Th17 cells and IL-7 promoted GM-CSF^+^ T cells represent functionally non-overlapping T cell subsets, we found that IL-23R expression was not induced upon IL-7-mediated Th_GM_ polarization *in vitro*. This result fits well to the established model that IL-23R expression is regulated downstream of STAT3 signaling, e.g., in response to IL-6 and IL-23 itself while IL-7-induced STAT5 signaling was not reported to impact IL-23R expression regulation (30). Furthermore, this finding provides mechanistic insight complementing our functional data showing that while IL-23R-expressing T cell subsets (i.e., Th17 cells) are critically promoting intestinal inflammation in syngeneic models of intestinal inflammation and in human IBD, allo-response driven colitis is mediated by IL-23R-, RORγt- and hence Th17 cell-independent effector T cells that require signals (i.e., IL-7/IL-7R interaction) clearly distinguishable from signals controlling Th17 cells (i.e., IL-23R/IL-23 signaling) and are characterized by the exertion of effector mechanisms (GM-CSF) clearly distinct from those employed by Th17 cells (i.e., IL-17a, etc.).

The overall observation that syngeneic colitis preferentially results in the formation of functionally central Th17 cells while Th_GM_ cells predominately develop and promote intestinal GvHD in the context of allo-reactivity is intriguing but the mechanisms underlying both the quantitative and qualitative differences remain elusive. However, following disease-related aspects might contribute to this phenomenon: first, although both syngeneic and allo-response-driven colitis formation depend on co-stimulatory signals that are provided by microbial components from colonizing microbiota (e.g., residing in the gut) and are also called pathogen-associated molecular pattern signals, host conditioning through whole body irradiation, etc., prior allo-HSCT results in a wide-spread tissue injury (39). In the course of the tissue response, a massive release of mediators like ATP or uric acid, also called danger-associated molecular pattern signals, takes place that additionally may account for the qualitative differences within colitogenic T cell pools observed during syngeneic versus allo-response driven colitis (40–43). Second, in addition to differential provision of co-stimulatory signals, setting-specific engagement of selective antigen-presenting cell subsets crucially impacts T cell differentiation outcomes. Accordingly it was shown that adoptively transferred T cells respond to microbial antigens presented by DCs (44) and CD64^+^CD11b^hi^ myeloid cells (45) and that their secretion of proinflammatory cytokines as IL-23 and IL-1β is critically contributing to the formation of colitogenic Th17 cells in the syngeneic colitis setting (46–48). By contrast, the expansion and differentiation of allo-reactive donor T cells were shown to be preferentially governed by donor-derived IL-12- and IL-6-secreting CD103^+^CD11b^−^ DCs (49). Since these studies did not investigate the impact on Th_GM_ cell formation, future studies need to investigate whether these DC subsets and DC-derived signals as suggested are acting in concert with IL-7 to drive the formation of intestinal GvHD-mediating GM-CSF expressing T cells.

Collectively, based on the large body of evidence in the literature RORγt-dependent Th17 cells are indispensable for the formation of a colitogenic T cell pool under syngeneic conditions (50). However, in the light of our current study results, we conclude that allo-response driven mucosal inflammation occurs in an IL-23R/Th17-independent, however, IL-7R/BATF-dependent manner and is mediated by GM-CSF rather than the classic Th17 signature cytokine IL-17a. Hence, we suggest that future attempts to limit GvHD-mediated mucosal inflammation especially affecting the colon should re-focus and evaluate the therapeutic efficacy of strategies designed to interfere with the development and/or functionality of GM-CSF expressing T cell subsets. However, one important question has not been answered yet: why and by what effector mechanisms exert Th17 cells colitogenecity in the syngeneic setting (11) but Th_GM_ cells (19) do not while as shown in this study Th_GM_ cells are highly pathogenic under allogeneic conditions whereas Th17 cells seem to be functionally dispensable? Hence, the solution of the major mechanistic conundrum overtly separating syngeneic from allogeneic colitis pathogenesis is reserved for future studies.

## Ethics Statement

This study was carried out in accordance with the recommendations of the government of Mittelfranken in Bavaria, Germany. The protocol was approved by the government of Mittelfranken in Bavaria, Germany. This study was carried out in accordance with the recommendations of the ethics committee of the University Hospital Regensburg. The protocol was approved by the ethics committee of the University Hospital Regensburg. All subjects gave written informed consent in accordance with the Declaration of Helsinki.

## Author Contributions

VB, BA, and JR performed experiments with the help of TV, analyzed and interpreted data. EH and SG provided human colon tissue samples, performed analyses related to human colon biopsies and helped with the interpretation and critical discussion of the results. MB-H performed histopathological analyses. MN, EU, and EH gave critical advice and helped with the interpretation and critical discussion of the results. KH directed the study and wrote the manuscript together with VB.

## Conflict of Interest Statement

The authors declare that the research was conducted in the absence of any commercial or financial relationships that could be construed as a potential conflict of interest.
