# Supplementary material for: Targeting Inflammatory T Helper Cells via Retinoic Acid-Related Orphan Receptor Gamma t Is Ineffective to Prevent Allo-Response-Driven Colitis
Source: Front Immunol. 2018 May 25;9:1138. doi: 10.3389/fimmu.2018.01138 (PMC5992389; doi:10.3389/fimmu.2018.01138)
Supplement: Supplementary file 1 [file Data_Sheet_1.docx]

Supplementary Material

Targeting Inflammatory T Helper Cells *via*
Retinoic Acid-Related Orphan Receptor Gamma t Is Ineffective to Prevent Allo-Response- Driven Colitis

Vera Buchele^1^, Benjamin Abendroth^1^, Maike Büttner-Herold^2^, Tina Vogler^1^, Johanna Rothamer^3,5^, Sakhila Ghimire^4^, Evelyn Ullrich^3,5^, Ernst Holler^4^, Markus F. Neurath^1^, Kai Hildner^1^*

^1^Department of Medicine 1, University Hospital Erlangen, University of Erlangen-Nuremberg, Kussmaul Campus for Medical Research, Erlangen, Germany

^2^Institute of Pathology, Department of Nephropathology, University Hospital Erlangen, Erlangen, Germany

^3^Department of Medicine 5, University Hospital Erlangen, University of Erlangen-Nuremberg, Erlangen, Germany

^4^Department of Hematology and Oncology, Regensburg University Hospital, Germany

^5^Childrens Hospital, Department of Pediatric Stem Cell Transplantation and Immunology, Johann Wolfgang Goethe University, Frankfurt, Germany

*** Correspondence:**Prof. Dr. med. Kai Hildner

Department of Medicine 1

University Hospital Erlangen

Ulmenweg 18

D-91054 Erlangen

Kai.Hildner@uk-erlangen.de

Phone ++49 9131 85 35 000

Fax ++49 9131 85 35 209

**
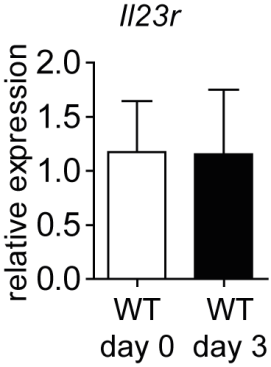
**

**Supplementary Figure 1.** **IL-7-induced Th5 cells do not upregulate *Il23r* gene expression.** Splenic CD4^+^ WT T cells were enriched by negative selection using magnetic microbeads followed by sort purification of naïve CD4^+^CD25^-^ T cells using flow cytometry. T cells were harvested directly after sort purification at day 0 (n=3) or after 3 days (n=3) of culturing under Th5 polarizing conditions (recombinant IL-7 and anti-IFNγ) and transcribed into cDNA followed by qPCR analyses of *Il23r* transcript levels. Gene expression levels detected within T cells harvested at day 0 were arbitrarily set down to 1 and all other gene expression levels were normalized to the expression level of this control. Data are combined from three individual experiments and were analyzed by Student’s *t* test. Data display mean values ±SEM.
